# Optimization of workflow for detection of brain metastases at 3T: is a black-blood MTC prepared 3D T1 used alone robust enough to replace the combination of conventional 3D T1 and the black-blood 3D T1 MTC?

**DOI:** 10.1007/s00234-023-03143-8

**Published:** 2023-03-30

**Authors:** Sophia Chkili, Yolène Lefebvre, Shih-Li Chao, Maria Antonietta Bali, Marc Lemort, Nicolas Coquelet

**Affiliations:** Department of Radiology, Institut Jules Bordet, 90 Rue Meylemeersch, 1070 Brussels, Belgium

**Keywords:** Brain metastases, Contrast enhancement, T1-weighted sequence, Magnetization transfer contrast, Black-blood, Vessel suppression

## Abstract

**Purpose:**

Sampling perfection with application-optimized contrasts by using different flip angle evolutions (SPACE) is a black-blood 3D T1-weighted (T1w) magnetic resonance imaging (MRI) sequence that has shown robust performance for brain metastases detection. However, this could generate false positive results due to suboptimal blood signal suppression. For that reason, SPACE is used in our institution alongside a non-black-blood T1w sequence: volumetric interpolated breath-hold examination (VIBE). Our study aims to (i) evaluate the diagnostic accuracy of SPACE compared to its use in combination with VIBE, (ii) investigate the effect of radiologist’s experience in the sequence’s performance, and (iii) analyze causes of discordants results.

**Methods:**

Four hundred seventy-three 3T MRI scans were retrospectively analyzed following a monocentric study design. Two studies were formed: one including SPACE alone and one combining both sequences (SPACE + VIBE, the reference). An experienced neuroradiologist and a radiology trainee independently reviewed the images of each study and reported the number of brain metastases. The sensitivity (Se) and specificity (Sp) of SPACE compared to SPACE + VIBE in metastases detection were reported. Diagnostic accuracy of SPACE compared to SPACE + VIBE was assessed by using McNemar’s test. Significance was set at *p* < 0.05. Cohen’s kappa was used for inter-method and inter-observer variability.

**Results:**

No significant difference was found between the two methods, with SPACE having a Se > 93% and a Sp > 87%. No effect of readers’ experience was disclosed.

**Conclusion:**

Independently of radiologist’s experience, SPACE alone is robust enough to replace SPACE + VIBE for brain metastases detection.

## Background

Brain metastases are the most common intracranial tumors in adults, affecting 20 to 30% of patients with cancer [1]. The most widespread primary tumors responsible for secondary cerebral lesions are lung cancer, breast cancer, and melanoma, with brain metastases occurring in approximately 40–50%, 15–20%, and 5–20% respectively [2], and melanoma having the highest predilection to metastasize to the brain [3, 4]. The incidence of brain metastases appears to be expanding over time as a result of an overall increase in primary cancers and better systemic therapies, which enhances the likelihood of metastatic disease as patients live longer [5]. Therefore, precise detection of intracranial secondary lesions is essential for adequate oncological treatment [1, 6]. This is all the more true regarding the exact number of metastases detected, given the fact that Gamma Knife stereotactic radiosurgery (GKSR) is now considered as the treatment of choice for patients with a limited number of brain metastases (for reviews, see e.g., [7, 8]). It is also crucial to better visualize small lesions, for which the GKSR will be proposed to the patient (usually a lesion below 2 cm^3^ or between 2 and 5 cm^3^) [9, 10].

Magnetic resonance imaging (MRI), particularly contrast-enhanced (CE) three-dimensional (3D) T1-weighted (T1w) imaging, is the standard of reference in brain metastases detection thanks to its excellent soft-tissue contrast [7, 8]. T1w sampling perfection with application-optimized contrasts by using different flip angle evolutions (SPACE, Siemens Healthineers, Erlangen, Germany) is a 3D turbo spin echo (TSE) sequence that is inherently black-blood (i.e., saturation of signal from blood vessels due to intravoxel dephasing among blood spins), and has the advantage of low specific absorption rate and high signal-to-noise ratio (SNR). The corresponding of the SPACE sequence for the other MRI constructors are CUBE for General Electrics, VISTA/BRAINVIEW for Philips, isoFSE for Hitachi, and 3D MOVX for Canon. Critically, as some slow flow distribution (e.g., in cortical veins) may be inaccurately suppressed, the SPACE sequence generally comes along with an additional black-blood preparation module (e.g., delay alternating with nutation for tailored excitation or DANTE) that optimizes blood-related signals suppression. A magnetization transfer preparation contrast (MTC) can also be added to the SPACE sequence to suppress the signal from background tissues and to render CE areas more conspicuous, enabling the sequence to be highly sensitive in detecting secondary brain lesions [9–12]. In fact, the combination of MTC and the use of paramagnetic contrast agents enables to augment the contrast between enhancing areas (i.e., intracerebral tumors) and surrounding tissue, and even more for enhancing tumors such as brain metastases [17].

In our imaging department, CE 3D T1w DANTE MTC SPACE (for convenience henceforth referred to as “SPACE” in this study) is used in routine screening and follow-up of brain metastases, alongside a 3D T1w gradient-recalled echo (GRE) sequence (volumetric interpolated breath-hold examination or VIBE, Siemens Healthineers, Erlangen, Germany) and 3D T2-weighted fluid attenuated inversion recovery (FLAIR). The rationale for using another CE T1w sequence (which does not have a preparation module for blood suppression) in addition to SPACE is guided by the reduction of false positive (FP) rate which might happen if SPACE was used alone. Indeed, it has been reported that black-blood techniques could insufficiently suppress signals from blood vessels and therefore mimic metastatic tumors [18]–[20]. Notwithstanding, in our institution, in order to shorten examination time, increase patient throughput, improve patient comfort, and reduce image interpretation time, we have considered the hypothesis of using the SPACE sequence as the sole T1w sequence in our imaging protocol for brain metastases evaluation.

The first aim of this study is therefore to assess diagnostic accuracy of SPACE compared to our current protocol (i.e., SPACE and VIBE) for brain metastases detection in order to assess if SPACE could be used alone, without combining it with the VIBE sequence. Our secondary endpoint is to evaluate the effect of the radiologist’s experience in diagnostic accuracy. Finally, the tertiary objective is to analyze and detail the main causes of discordant results, namely, FP and false negatives (FN), in brain metastases detection by using SPACE alone.

## Methods

### Study design and population

This study was approved and waived for patients’ consents by the ethics committee of the institution. This monocentric retrospective study includes all patients who underwent cerebral MRI scans for screening and follow-up of brain metastases, between 29/09/2018 and 09/11/2019 on a 3 tesla (3T) magnetic field scanner. This timeframe represents about 500 MRI examinations. Importantly, all patients presented known primitive tumors and were referred for an MRI brain scan either for a routine follow-up or the investigation of neurological symptoms. We subsequently extracted from the full routine protocol only VIBE and SPACE sequences. On this basis, two studies were formed: one including VIBE and SPACE images (henceforth referred to as “SPACE + VIBE”) and the other SPACE images only. Studies were randomized differently, and patients were anonymized. Eleven MRI scans were excluded due to absence of contrast administration, either because of previous hypersensitivity reactions to gadolinium-based contrast agents or severely impaired kidney function. During imaging interpretation, 16 MRI scans were discarded because the quality was deemed insufficient: 7 due to artifacts hindering optimal interpretation and 9 due to incomplete acquisition. Taken together, a total of the 473 remaining scans were analyzed. Sex, age at MRI examination and primary tumor were extracted from the institute’s medical file database.

### Imaging acquisition technique

All examinations were performed at the 3T magnetic field scanner MAGNETOM Skyra fit® (Siemens Healthineers, Erlangen, Germany). A gadolinium-based contrast agent — GADOVIST® 1.0 mmol/ml (Bayer Schering Pharma AG, Berlin, Germany) — was injected at least 10 min before data acquisition at a dose of 0.1 mmol/kg.

The SPACE sequence was acquired before the VIBE sequence with the following parameters: repetition time (TR) and echo time (TE) being TR/TE = 643/21 ms, resolution = 0.8 mm^3^ (isometric), acquisition time: 4 min 48. Specific features of SPACE were the variable flip angle evolution intended for short TE application, the DANTE dark-blood preparation, slab selective excitation in the axial plane and added MTC preparation. The VIBE sequence was performed afterwards, with the following parameters: TR/TE = 4.7/1.76 ms, resolution = 0.8 mm^3^ (isometric), leading to an acquisition time: 3 min 23.

### Image analysis

Two investigators, an experienced radiologist (ER) with 15 years of experience in neurological imaging and a junior radiologist (JR) with a 5-year experience in general radiology, independently analyzed both studies using the platform Telemis-Medical® Version 4.95 (Ottignies-Louvain-la-Neuve, Belgium). Investigators were blinded regarding the patient’s identity and the primary tumor diagnosis. However, they were not blinded to the sequence type due to its distinctive appearance. Within each set of sequences, readers reported the number of metastases (ranging from 0 to > 10; 0 meaning the absence of metastases and > 10 that more than 10 metastases were found). To avoid recall biasing of the observers, the reading sessions of both studies were separated by an interval of at least 6 weeks.

### Statistical analysis

Statistical analyses were performed using MatlabR2022.

Non-parametric McNemar’s tests were used to assess diagnostic accuracy between SPACE and SPACE + VIBE (this latter being the standard of reference chosen for this study) in detecting brain metastases. Tables of contingency, including true positives (TP), true negatives (TN), FP, and FN, were constructed. Sensitivity (Se), specificity (Sp), positive predictive value (PPV), and negative predictive value (NPV) of each investigated case were then calculated and reported within their 95% confidence interval (CI). Non-parametric Spearman correlation tests were used to assess statistical differences in the number of brain metastases detected by both JR and ER, for both SPACE and SPACE + VIBE sequences. Differences were deemed significant whenever the corresponding *p*-value was lower than 0.05. Finally, Cohen’s kappa (κ) method was used to evaluate the inter-observer and inter-method reliability. Following [21], κ values ranging between 0.8 and 1 indicate an almost perfect agreement.

### Descriptive analysis of discordant cases

All cases of discordance (i.e., FP and FN) were extracted and analyzed individually. Each discordant case was examined a second time by studying both sequences in conjunction and trying to find explanations for their erroneous assignment. The corresponding T2w FLAIR sequence was also taken into consideration whenever deemed useful. Importantly, this part of the analysis was neither randomized nor anonymized, and was carried out by the JR and the ER, in consensus with a third party (an experienced neuroradiologist with over 30 years of experience in neurological imaging).

## Results

### Study population characteristics

Four hundred seventy-three MRI examinations from 292 patients (mean age ± standard deviation (SD): 62 ± 11, range: 31–89 years, 296 females and 177 males) were included in our study. Main primary neoplasms are represented by lung cancer (196/473, mean age ± SD: 65 ± 9), breast cancer (140/473, mean age ± SD: 58 ± 11 years) and melanoma (82/473, mean age ± SD: 65 ± 9 years). Other primary localizations (55/473) include renal carcinoma, colorectal cancer, anal cancer, ovarian cancer, neuroendocrine tumors, uterine cancer, germinal cell carcinoma, gastric cancer, gastrointestinal stromal tumor, leukemia, mediastinal adenocarcinoma, nasopharyngeal cancer, urothelial carcinoma, sinonasal undifferentiated carcinoma, chondrosarcoma, osteotropic cancer, prostate cancer, sarcoma, squamous cell carcinoma, and testicular cancer.

### Diagnostic performance of SPACE in brain metastases detection

**Fig. 1:**
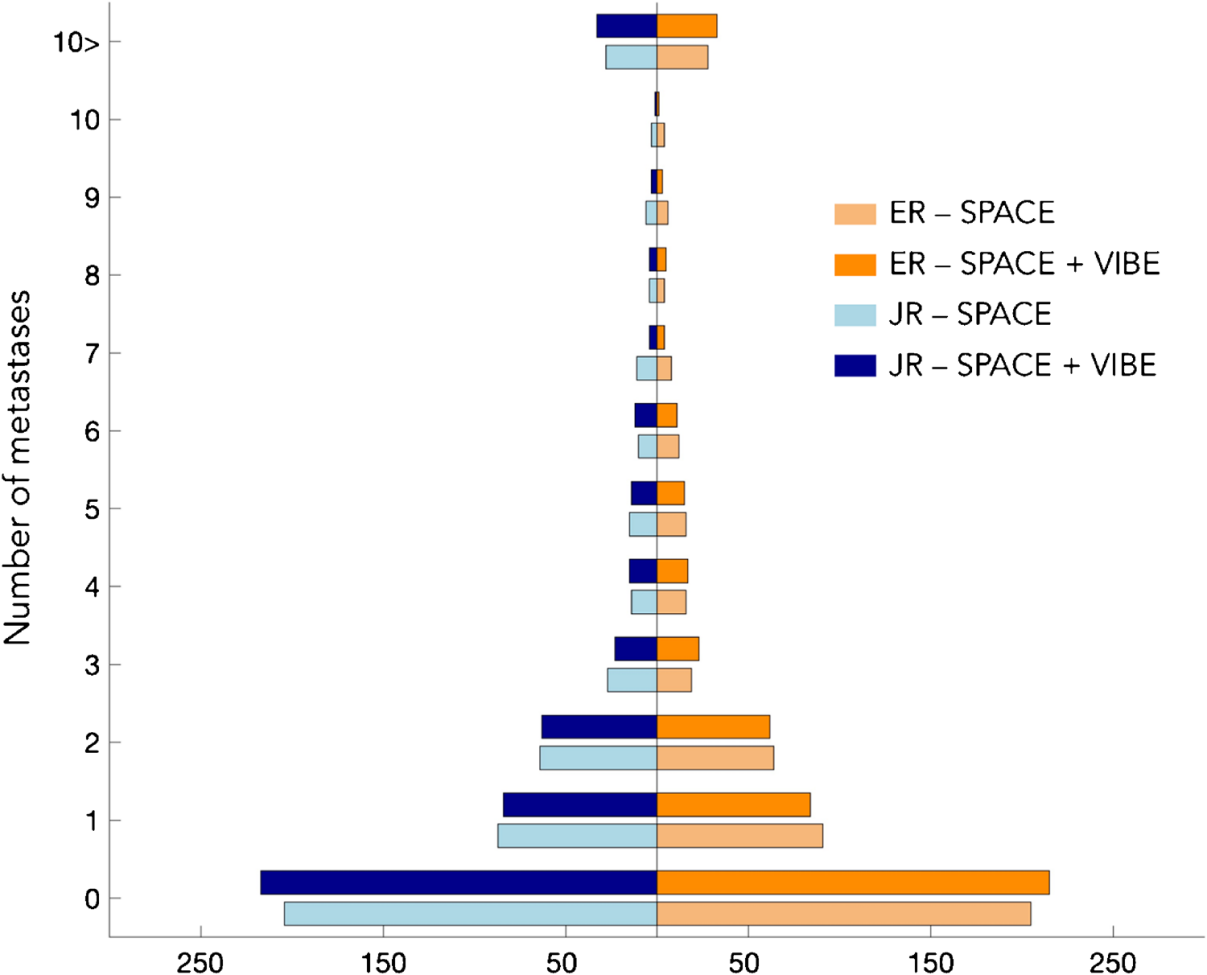
Distribution of the number of brain metastases for the JR (left) and ER (right). Lighter colors (i.e., light orange and light blue) refer to detection of metastases relying on SPACE sequence only and darker colors (i.e., dark orange and dark blue) are associated with detection based on SPACE + VIBE sequences. SPACE, sampling perfection with application-optimized contrasts by using different flip angle evolutions; VIBE, volumetric interpolated breath-hold examination; ER, experienced radiologist; JR, junior radiologist.

Figure 1 shows the histogram of the number of brain metastases detected using either SPACE or SPACE + VIBE for the JR and ER.

The table of contingency displaying the number of parenchymal brain lesions using SPACE for both junior and experienced readers is reported in Table 1. To note, as aforementioned, SPACE + VIBE was taken as reference.

**Table 1:** Diagnostic performance of SPACE compared to SPACE + VIBE in brain metastases detection. TP, TN, FP, FN, Se, Sp, PPV, NPV, *p*-value, and Cohen’s κ value are reported for both the JR (first row) and the ER (second row). 95% confidence intervals are expressed within parenthesis

	TP	TN	FP	FN	Se (%)	Sp (%)	PPV (%)	NPV(%)	*p*-val	κ
JR	189	189	28	15	92.6 (88.1–95.8)	12.9 (8.7–18.1)	87.1 (81.9–91.2)	92.6 (88.1–95.8)	0.05	0.82
ER	241	188	27	17	93.4 (89.6–96.1)	12.5 (8.4–17.7)	89.9 (85.7–93.3)	91.7 (87.1–95.1)	0.13	0.81

*SPACE*, sampling perfection with application-optimized contrasts by using different flip angle evolutions; *VIBE*, volumetric interpolated breath-hold examination; *TP*, true positives; *TN*, true negatives; *FP*, false positives; *FN*, false negatives; *Se*, sensitivity; *Sp*, specificity; *PPV*, positive predictive value; *NPV*, negative predictive value; *ER*, experienced radiologist; *JR*, junior radiologist

These contingency tables show that the detection of brain metastases can be similarly performed using either the SPACE sequence alone or in combination with the VIBE sequence (i.e., SPACE + VIBE) (no statistical difference, *p* > 0.05). These results hold true for both ER and JR.

### Diagnostic performance of SPACE in number of metastases detected

Correlation tests show no difference in the number of brain metastases (correlation coefficient ⍴ > 0.88, *p* < 0.001) between SPACE and SPACE + VIBE sequences for fixed radiologist (see rows 1 and 2 of Table 2) and between radiologists for fixed sequence (see rows 3 and 4 of Table 2).

**Table 2:** Correlation coefficient and associated *p*-value for the number of metastases

		Correlation (95% CI) *p*-value
ER / SPACE	ER / SPACE + VIBE	0.88 (0.85–0.9) *p* < 0.001
JR / SPACE	JR / SPACE + VIBE	0.88 (0.85–0.9) *p* < 0.001
ER / SPACE	JR / SPACE	0.99 (0.99–0.99) *p* < 0.001
ER / SPACE + VIBE	JR / SPACE + VIBE	0.99 (0.99–0.99) *p* < 0.001

*CI*, confidence interval; *SPACE*, sampling perfection with application-optimized contrasts by using different flip angle evolutions; *VIBE*, volumetric interpolated breath-hold examination; *ER*, experienced radiologist; *JR*, junior radiologist

### Interobserver variability

Interobserver variability assessed by Cohen’s κ test shows an overall excellent agreement (i.e., Cohen’s κ value ranging between 0.8 and 1) between ER and JR in the evaluation of the presence or absence of metastases both with SPACE (κ = 0.99, CI: 0.97–1) and SPACE + VIBE (κ = 0.98, CI: 0.97–1)

### Detailed analysis of discordant results

Together, we have identified 28 FP cases for the JR and 27 for the ER (with an overlap of 26 cases between the readers), and 15 FN cases for the JR and 17 for the ER (with on overlap of 14 cases between the readers). These cases of discordance are more extensively discussed hereunder.

#### False positives

**Fig. 2:**
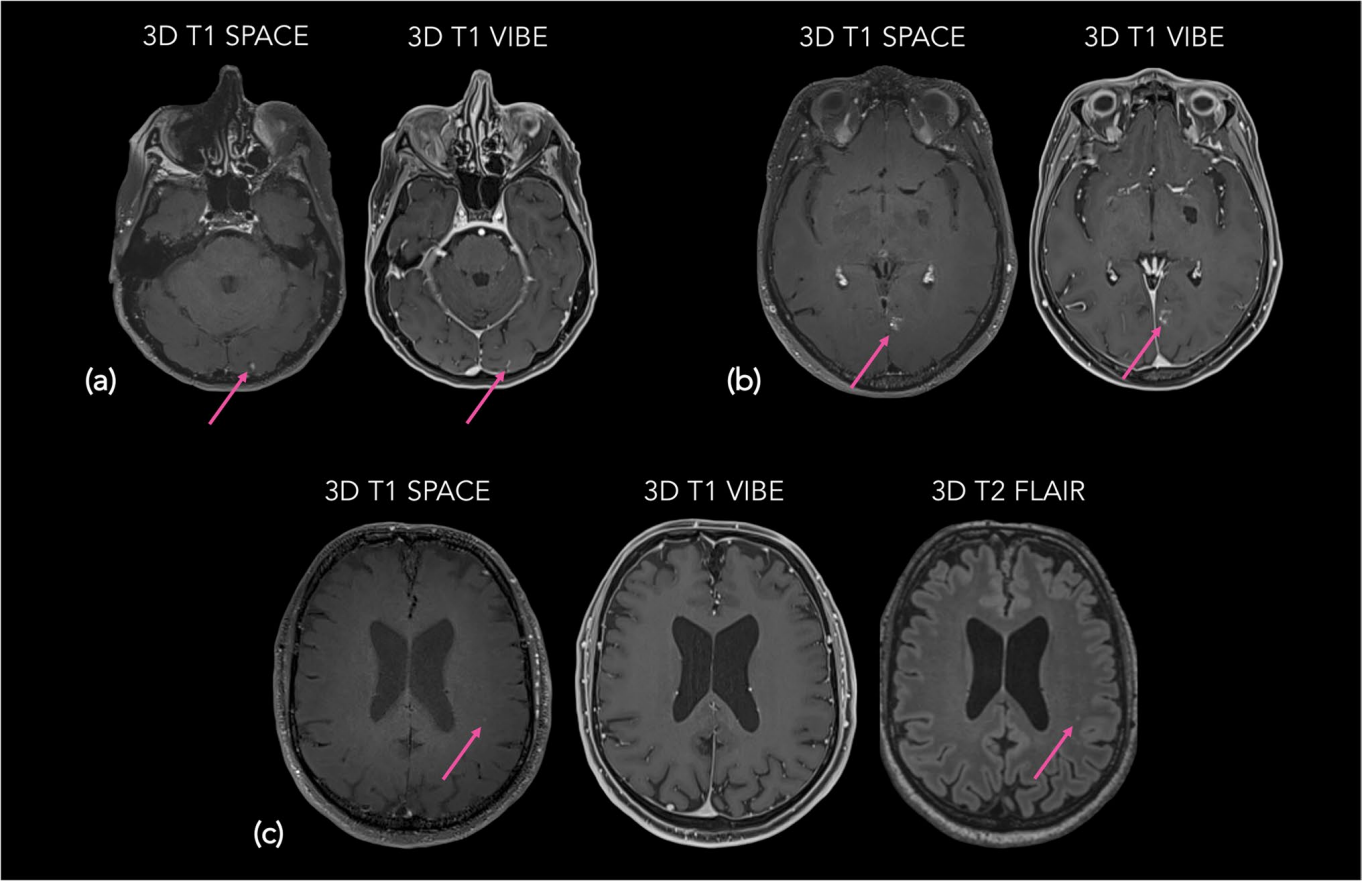
Examples of false positive cases of SPACE in detecting brain metastases demonstrated by CE axial slices. **a** Non-suppressed small left occipital cortical vessel (arrow) appearing on SPACE (left) as an enhancing lesion, better discernible on VIBE (right) in a 70-year-old female with lung cancer. **b** Non-suppressed left parasagittal developmental venous anomaly (arrow) appearing on SPACE (left) as an enhancing lesion, better discernible on VIBE (right) in a 62-year-old female with lung cancer. **c** Left periventricular white matter hyperintensity (arrow) appearing on SPACE (left) as an enhancing lesion; confirmed in T2w FLAIR sequence (right); no enhancing area is visible on VIBE (middle) in a 65-year-old male with lung cancer. SPACE, sampling perfection with application-optimized contrasts by using different flip angle evolutions; CE, contrast-enhanced; VIBE, volumetric interpolated breath-hold examination; T2w, T2 weighted; FLAIR, fluid attenuated inversion recovery; 3D, three dimensional

Eight FP for the JR and 4 for the ER were due to detection errors (distraction errors, small lesion < 2 mm, peripheral topography). Seven FP for the JR and 10 for the ER were due to incomplete vessel suppression. In 6 cases for the JR and 2 for the ER, contrast enhancements in the rim of metastasis resection cavities were wrongly considered as lesions. Four FP for the JR and 1 for the ER were due to small leptomeningeal enhancement considered as parenchymal lesions. Two FP (2 for each reader) were caused by remaining artifacts that were not excluded during the first study selection. Three FP were due to diagnosis errors made by ER (ischemic stroke lesions for instance). One FP for the JR and 3 for the ER were attributed to retranscription mistakes. Lastly, 2 FP for ER were due to hyperintense areas caused by leucopathy. Illustrative examples of FP are displayed in Fig. 2.

#### False negatives

**Fig. 3:**
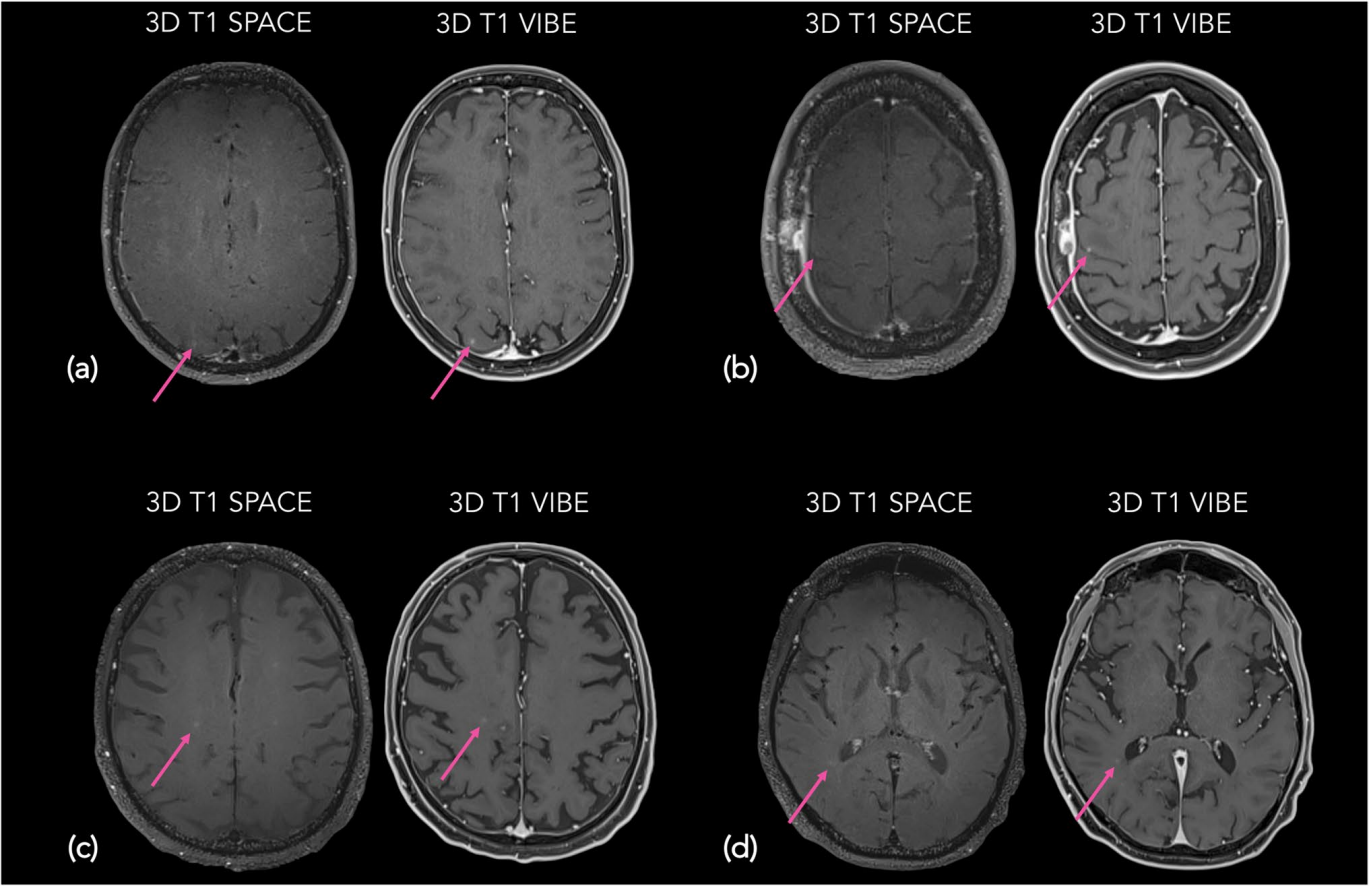
Examples of false negative cases of SPACE in detecting brain metastases demonstrated by CE T1w axial slices. **a** Very small (< 2 mm) cortical right occipital peripheral lesion (arrow) missed on SPACE (left), better discernible on VIBE (right) in a 59-year-old female with lung cancer. **b** Very small (2 mm) para-sulcal right frontal lesion (arrow) missed on SPACE (left), better discernible on VIBE (right) in a 59-year-old female with lung cancer. **c** Poorly enhancing right parietal metastasis (arrow) wrongly considered as non-attenuated white matter hyperintensity on SPACE (left), confirmed as such on VIBE (right) in a 70-year-old male with lung cancer. **d** Poorly enhancing right parietal periventricular metastasis (arrow) wrongly considered as non-attenuated white matter hyperintensity on SPACE (left), confirmed as such on VIBE (right) in a 71-year-old male with lung cancer. SPACE, sampling perfection with application-optimized contrasts by using different flip angle evolutions; CE, contrast-enhanced; VIBE, volumetric interpolated breath-hold examination; T1w, T1 weighted; 3D, three dimensional

Five FN for the JR and 9 for the ER were due to detection errors (distraction errors, small lesion < 2 mm, trapping topography). Three FN for the JR and 2 for the ER were due to low-intensity enhancing metastases that were wrongly considered as hyperintensities caused by leucopathy. Three FN for the JR and one for the ER were a consequence of artifacts. Two cases (2 for each reader) were due to small lesions in or next to enhancing resection cavities. One FN for the JR and 2 for the ER were attributed to confusion between leptomeningeal enhancement and parenchymal enhancement. One FN for JR was due to a metastatic lesion considered as a non suppressed vessel in SPACE. Lastly, 1 FN for the ER is because of a retranscription mistake. Illustrative examples of FN are shown Fig. 3.

## Discussion

The purpose of our study was threefold: (1) to investigate whether the sole use of SPACE is sufficient in detecting brain metastases instead of using it in combination with a classical GRE non black-blood sequence (here VIBE); (2) the effect of radiologist’s experience on this potential difference; and (3) the causes of discordant results in SPACE’s performance for brain metastases detection.

Our results highlighted that SPACE alone is robust enough in detecting brain metastases as no statistical difference with the use of SPACE and VIBE together was observed. In addition, we found no effect of the radiologist's experience, with the junior reader performing as well as the experienced one. The main causes of FP and FN results were also identified. Importantly, analyses were re-conducted for each primary cancer subtype and no statistical effect of the primary cancer subtype was found (data not shown).

To the best of our knowledge, this is the first study that compares the use of SPACE alone to the association SPACE + VIBE in intracerebral secondary lesions assessment. Our study showed high Se, Sp, PPV, and NPV for SPACE in detecting intraparenchymal metastatic lesions regardless of the investigator’s experience. In that framework, our study is in line with Kikuchi et al. [22] who found close results (i.e., no effect of radiologist’s experience) comparing a similar sequence called volume isotropic simultaneous interleaved bright- and black-blood examination (VISIBLE) with a conventional GRE imaging. Of note, the result of our study would have been strengthened with additional readers but our results, together with those of Kikuchi et al. [22], suggest that the radiologist’s experience remains marginal for brain metastases detection.

Numerous studies (e.g., [9, 12]) have compared a 3D TSE sequence similar to SPACE with other T1w CE sequences, principally with magnetization-prepared rapid gradient-echo (MPRAGE) due to its widespread use in clinical routine. In fact, 3D GRE sequences such as MPRAGE were historically developed before 3D TSE and were thus used first for brain metastases detection. The main differences between 3D TSE and 3D GRE are (i) white and gray matter contrast (better for 3D GRE), (ii) blood flow signals (hypointense for 3D TSE, hyperintense for 3D GRE), and (iii) magnetic susceptibility artifacts (reduced for 3D TSE, present for 3D GRE) (for a review, see, e.g., [23]). Notwithstanding these differences, studies have shown that 3D TSE has better detection rate of brain metastases compared to 3D GRE (e.g., [13, 24]). Our study emphasizes that those high detection rates are still valid even when comparing SPACE to a conjunction of two highly accurate sequences, herein SPACE and VIBE.

Although there was no significant statistical difference between SPACE alone and the combination of SPACE and VIBE, the rate of brain metastases detection was not perfect as some FP and FN results were still observed. Therefore, we leaned on analyzing each of those cases to better comprehend remaining weaknesses linked to the use of SPACE alone. To note, it should be underscored that, for the discordant cases, the consensus reading among the two radiologists (and the third party) was taken as the standard of reference. In our series, 30% of FP were due to suboptimal blood vessel signal suppression, which is far less than the 70% rate reported by Nagao et al. [18] who studied a TSE black-blood sequence (called MSDE) versus the MPRAGE sequence. This suggests that the addition of the DANTE blood signal suppression preparation to our SPACE sequence, which already contains an intrinsic blood-suppression characteristic, is critical to improve vessel signal suppression. The study of Xie et al. [19] shows the same results for intracranial vessel wall imaging. Another cause of FP or FN that has been highlighted in our study comes from periventricular white matter hyperintensities, also known as leukoaraiosis. In fact, these latter are striking abnormalities, often disclosed on T2w and FLAIR images in the elderly population [25]. They occur in about 30% of healthy subjects over 60 years of age [26]. Importantly, our SPACE sequence was further improved by the addition of a MTC preparation to attenuate the white matter signal surrounding enhancing brain lesions making them easily discernible. Due to these magnetization transfer properties, regions affected by white matter loss, corresponding from a molecular point of view to the loss of axons and myelin sheaths (that is, white matter hyperintensities) [27], were not properly attenuated and therefore remained hyperintense on SPACE, and potentially misleading the reader in taking them for metastases. The main parameter implicated in this effect is the magnetization transfer ratio (MTR) that measures the efficiency of the magnetization exchange between the relatively free water protons inside tissue and those bound to protein macromolecules in cellular membranes. A reduction of the MTR is seen in all pathological changes in brain tissue structure that involve alterations in cell membrane macromolecules, such as inflammation, myelin pallor or demyelination [21–23]. In this further analysis of FP and FN cases, we had to resort to T2w FLAIR imaging — which is considered to be the sequence of reference for studying white matter changes [31] — to differentiate the “real” enhancing lesions from non-attenuated white matter in leukoaraiosis. This to say that a T2w FLAIR sequence is key for adequate interpretation of MRI images in brain metastases evaluation, especially in imaging protocols resorting to MTC prepared T1w sequences. This also holds true for leptomeningeal secondary involvement, a less frequent but yet important part of brain metastatic disease [32]. Despite some inadequate attenuations of white matter signals with the MTC preparation module as discussed hereinabove, the key role of this latter module should be underlined for a correct assessment of brain metastases. To our view, we claim for this module to be constantly added to the SPACE sequence to enhance brain metastases visualization. Finally, some of the lesions missed by radiologists using SPACE alone were due to their small size. Nonetheless, high detection rates in black-blood TSE T1w sequences with variable flip angles (such as SPACE) are still well established in literature [9, 12], mostly because of a significantly better contrast to noise ratio (CNR) of tumor to parenchyma. This is especially true for very small lesions (inferior to 5 mm) [33]. Kim et al. [34] reported approximately double the detection rates of ≤ 5 mm brain metastases with a DANTE-SPACE compared with MPRAGE as well as higher CNR of metastases and shorter reading time. This contrasts with our study where all the small lesions missed were inferior or equal to 2 mm. Detection of those very small lesions is crucial for treatment management (e.g., for GKSR for instance) [13]. Despite this, data on variability of measurements and localizations of brain metastases are still limited to date [35].

This study suffers some limitations. In addition to the design being retrospective and monocentric, one of our limitations is that it included only MRI scans carried out on a 3T magnetic field system. This choice was guided by the better SNR and the higher resolution of studies performed on 3T compared to 1.5T examinations in cerebral tumors [29–31]. Although most MRI scans are carried out on 3T in our institution, brain MRI scans on 1.5T systems remain inevitable for some patients due to technical or logistical reasons. Moreover, 3T scans are not available in all imaging centers. Therefore, it would be interesting to adapt our study to 1.5T MRI scans. Furthermore, we do recognize that our standard of reference, being only a part of the current protocol used in our department, is imperfect. A stronger — but rather impossible — referential would have been anatomopathological confirmation of metastatic brain lesions. However, histology was only acquired from the primary tumor, since patients with brain metastases generally do not undergo cerebral biopsy at our institution, unless primitive cancer is unknown. Another standard of reference used in several (longitudinal) studies is the follow-up. Although interesting, this standard of reference could not have been implemented herein as lots of patients came only once during the time study window (e.g., a screening that showed no presence of metastases). A last weakness of this study concerns the third part of the analysis in which discordant cases were taken out of the anonymized and randomized series and were interpreted in a real clinical setting. Finally, this study was a cross-sectional comparison for diagnostic accuracy, and its consequences on patients’ clinical outcome remain to be evaluated.

## Conclusions

In summary, we successfully demonstrated that SPACE, a 3D TSE black-blood with MTC preparation, is robust enough to replace the combination of two different T1w sequences in brain metastases detection, regardless of the radiologist’s experience. Therefore, for imaging centers with high patient flow and seeking for rapid imaging results, imaging protocol for brain metastases evaluation could be shortened, with only two contrast enhanced 3D sequences: a T1w sequence such as SPACE and a T2w sequence such as FLAIR, without loss of imaging quality and interpretation allowing rapid oncological care.

## Data Availability

The dataset analyzed in this study is available to the corresponding author on reasonable request after approval by the institution ethics committee.

## References

[1] Kaal ECA, Niël CGJH, Vecht CJ (2005). Therapeutic management of brain metastasis. Lancet Neurol.

[2] Eichler AF, Plotkin SR (2008). Brain metastases. Curr Treat Options Neurol.

[3] Amer MH, Al-Sarraf M, Baker LH, Vaitkevicius VK (1978). Malignant melanoma and central nervous system metastases: incidence, diagnosis, treatment and survival. Cancer.

[4] O’Neill BP, Buckner JC, Coffey RJ, Dinapoli RP, Shaw EG (1994). Brain metastatic lesions. Mayo Clin Proc.

[5] Soffietti R (2017). Diagnosis and treatment of brain metastases from solid tumors: guidelines from the European Association of Neuro-Oncology (EANO). Neuro Oncol.

[6] Ranjan T, Abrey LE (2009). Current management of metastatic brain disease. Neurotherapeutics.

[7] Kocher M (2014). Stereotactic radiosurgery for treatment of brain metastases. A report of the DEGRO Working Group on Stereotactic Radiotherapy. Strahlenther Onkol.

[8] Mehta MP (2005). The American Society for Therapeutic Radiology and Oncology (ASTRO) evidence-based review of the role of radiosurgery for brain metastases. Int J Radiat Oncol Biol Phys.

[9] Yomo S, Hayashi M, Nicholson C (2012). A prospective pilot study of two-session Gamma Knife surgery for large metastatic brain tumors. J Neurooncol.

[10] Ito D, Aoyagi K, Nagano O, Serizawa T, Iwadate Y, Higuchi Y (2020). Comparison of two-stage Gamma Knife radiosurgery outcomes for large brain metastases among primary cancers. J Neurooncol.

[11] Schellinger PD, Meinck HM, Thron A (1999). Diagnostic accuracy of MRI compared to CCT in patients with brain metastases. J Neurooncol.

[12] Sze G, Soletsky S, Bronen R, Krol G (1989). MR imaging of the cranial meninges with emphasis on contrast enhancement and meningeal carcinomatosis. AJR Am J Roentgenol.

[13] Komada T (2008). Contrast-enhanced MR imaging of metastatic brain tumor at 3 tesla: utility of T(1)-weighted SPACE compared with 2D spin echo and 3D gradient echo sequence. Magn Reson Med Sci.

[14] Park YW (2021). Robust performance of deep learning for automatic detection and segmentation of brain metastases using three-dimensional black-blood and three-dimensional gradient echo imaging. Eur Radiol.

[15] S. Yang, Y. Nam, M. O. Kim, E. Y. Kim, J. Park, and D. Kim, ‘Computer-aided detection of metastatic brain tumors using magnetic resonance black-blood imaging’, Investigative radiology, vo 48 2 2013, 10.1097/RLI.0b013e318277f078. 10.1097/RLI.0b013e318277f078 23211553

[16] Yoneyama M (2014). Improvement of T1 contrast in whole-brain black-blood imaging using motion-sensitized driven-equilibrium prepared 3D turbo spin echo (3D MSDE-TSE). Magn Reson Med Sci.

[17] Kurki TJ, Niemi PT, Lundbom N (1992). Gadolinium-enhanced magnetization transfer contrast imaging of intracranial tumors. J Magn Reson Imaging.

[18] Nagao E (2011). 3D turbo spin-echo sequence with motion-sensitized driven-equilibrium preparation for detection of brain metastases on 3T MR imaging. AJNR Am J Neuroradiol.

[19] Xie Y, Yang Q, Xie G, Pang J, Fan Z, Li D (2016). Improved black-blood imaging using DANTE-SPACE for simultaneous carotid and intracranial vessel wall evaluation. Magn Reson Med.

[20] Fu Q (2022). Comparison of contrast-enhanced T1-weighted imaging using DANTE-SPACE, PETRA, and MPRAGE: a clinical evaluation of brain tumors at 3 Tesla. Quant Imaging Med Surg.

[21] Landis JR, Koch GG (1977). The measurement of observer agreement for categorical data. Biometrics.

[22] Kikuchi K (2015). 3D MR sequence capable of simultaneous image acquisitions with and without blood vessel suppression: utility in diagnosing brain metastases. Eur Radiol.

[23] Bapst B (2020). Post-contrast 3D T1-weighted TSE MR sequences (SPACE, CUBE, VISTA/BRAINVIEW, isoFSE, 3D MVOX): technical aspects and clinical applications. J Neuroradiol.

[24] Danieli L (2019). Brain tumor-enhancement visualization and morphometric assessment: a comparison of MPRAGE, SPACE, and VIBE MRI techniques. AJNR Am J Neuroradiol.

[25] Spilt A, Goekoop R, Westendorp RGJ, Blauw GJ, de Craen AJM, van Buchem MA (2006). Not all age-related white matter hyperintensities are the same: a magnetization transfer imaging study. AJNR Am J Neuroradiol.

[26] Meyer JS, Kawamura J, Terayama Y (1992). White matter lesions in the elderly. J Neurol Sci.

[27] Bastin ME, Clayden JD, Pattie A, Gerrish IF, Wardlaw JM, Deary IJ (2009). Diffusion tensor and magnetization transfer MRI measurements of periventricular white matter hyperintensities in old age. Neurobiol Aging.

[28] Filippi M, Rocca MA (2007). Magnetization transfer magnetic resonance imaging of the brain, spinal cord, and optic nerve. Neurotherapeutics.

[29] Seiler S (2014). Magnetization transfer ratio relates to cognitive impairment in normal elderly. Front Aging Neurosci.

[30] Silver NC (1997). Magnetisation transfer ratio measurement in the cervical spinal cord: a preliminary study in multiple sclerosis. Neuroradiology.

[31] R. Sharma, S. Sekhon, and M. Cascella, ‘White matter lesions’, in *StatPearls*, Treasure Island (FL): StatPearls Publishing, 2022. Accessed: Jan. 17, 2023. [Online]. Available: http://www.ncbi.nlm.nih.gov/books/NBK562167/

[32] Singh SK, Leeds NE, Ginsberg LE (2002). MR imaging of leptomeningeal metastases: comparison of three sequences. AJNR Am J Neuroradiol.

[33] Suh CH, Jung SC, Kim KW, Pyo J (2016). The detectability of brain metastases using contrast-enhanced spin-echo or gradient-echo images: a systematic review and meta-analysis. J Neurooncol.

[34] Kim D (2019). Usefulness of the delay alternating with nutation for tailored excitation pulse with T1-weighted sampling perfection with application-optimized contrasts using different flip angle evolution in the detection of cerebral metastases: comparison with MPRAGE imaging. AJNR Am J Neuroradiol.

[35] Bauknecht H-C (2010). Intra- and interobserver variability of linear and volumetric measurements of brain metastases using contrast-enhanced magnetic resonance imaging. Invest Radiol.

[36] Le Rhun E (2017). ‘EANO-ESMO Clinical Practice Guidelines for diagnosis, treatment and follow-up of patients with leptomeningeal metastasis from solid tumours. Ann Oncol.

[37] Orbach DB (2006). Comparing real-world advantages for the clinical neuroradiologist between a high field (3 T), a phased array (1.5 T) vs. a single-channel 1.5-T MR system. J Magn Reson Imaging.

[38] Schwindt W (2003). Magnetic resonance imaging protocols for examination of the neurocranium at 3 T. Eur Radiol.

